# The epididymal sperm viability, motility and DNA integrity in dead mice maintained at 4-6oC

**Published:** 2013-03

**Authors:** Farhad Golshan Iranpour, Mojtaba Rezazadeh Valojerdi

**Affiliations:** 1 *Department of Biomedical Sciences, Anatomical Sciences Section, Isfahan University of Medical Sciences, Isfahan, Iran.*; 2 *Department of Anatomy, Shahrekord University of Medical Sciences, Shahrekord, Iran.*; 3 *Department of Embryology, Royan Institute, Tehran, Iran.*

**Keywords:** *DNA*, *Epididymides*, *Mouse*, *Spermatozoa*

## Abstract

**Background: **When male animals die, spermatozoa within the body of animal will be degenerated. Because of unique chromatin structure of sperm, maybe this degeneration is different from other cells. However there is not any research which considered directly the integrity of sperm DNA by keeping the cadaver in refrigerator.

**Objective:** The aim of this study was to assess viability, total motility and DNA integrity of sperm cells after death.

**Materials and Methods:**In this experimental study, 24 male Swiss white mice were killed by cervical dislocation and then kept in refrigerator (4-6^o^C) for up to 12 days. On the 0 (immediately after death as control group), 1^st^, 2^nd^, 3^rd^, 5^th^, 7^th^, 10^th^ and the 12^th^ days after death cauda epididymides were removed and squeezed in Ham’s F10 medium. The proportion of viable, motile and double stranded DNA spermatozoa was examined. Viability and DNA integrity of sperm cells were examined consecutively by eosin nigrosin and acridine orange stainings.

**Results:**The data obtained from this study showed that viability and total motility of sperm cells were significantly decreased during 12 days after death (p<0.001). In contrast with viability and motility, DNA integrity was without significant changes (even 12 days after death).

**Conclusion:**This study suggests that integrity of sperm DNA would not change even after 12 days after death if the cadaver kept in refrigerator.

## Introduction

It’s apparent that spermatozoa within the body of dead animals would eventually degenerate. However live spermatozoa have collected from mouse cadavers that have been maintained at room temperature up to 24 hours and these spermatozoa are able to fertilize at least 30% of oocytes in vitro (1). Also these sperm cells can be used to fertilize oocytes and the resulting zygotes can develop into live youngs (2). 

An *et al* maintained mice in refrigerator for various periods and examined viability of the epididymal spermatozoa by in vitro fertilization, embryo culture and embryo transfer. They found that spermatozoa from ICR(imprinting control region) male mice could fertilize 21% of oocytes after being stored at 4-6^o^C for 5 days and spermatozoa from BDF1 male mice(kind of hybrid mouse) could fertilize 39% of oocytes after being stored at 4-6^o^C for 7 days (3). Kishikawa *et al* examined motility, viability and fertility of mouse spermatozoa retrieved from mice cadavers that had been maintained at 4^o^C. They showed that about 30% of spermatozoa collected 10 days after death were viable, but they had limited ability to fertilize oocytes in vitro. Meanwhile if the spermatozoa were injected into oocytes, the fertilization rate was over 80%. Besides normal live fetuses can be obtained even by using of immotile spermatozoa retrieved 20 days after death (4). 

Kaneko and Nakagata showed that cauda epididymal spermatozoa of mouse that were freeze dried and then kept in 4^o^C for 5 months could fertilize oocytes and these oocytes developed to normal offsprings (suppression of nucleases present in the spermatozoa during storage led to achievement of long term storage of freeze dried spermatozoa) (5). 

Ward *et al* reported that both freeze dried (sperms kept at 4^o^C) and freezing of mouse sperm when used for ICSI (intracytoplasmic sperm injection) had similar results (about 87% of resultant zygotes contained normal chromosomes) (6). In the present study Swiss white mouse spermatozoa collected from epididymes till 12 days after their death (cadavers were stored at 4^o^C), were examined for their motility, viability and double stranded DNA.

Denaturation of DNA, that is the splitting of double stranded DNA into two single strands, can be induced by various factors. Theoretically after death, nuclear DNA will denature. For the assessment of sperm DNA, acridine orange (AO) stain was used. The aim of this study was to survey the changes of some sperm parameters such as motility, viability and DNA integrity during 12 days after death of animals. Although other researchers examined motility, viability and fertility of mouse spermatozoa but they didn’t examined the integrity of DNA after death. Because of unique packaging of sperm chromatin the degeneration of its nucleous maybe is different from other cells. This information can be very advantageous in case of unexpected death of a genetically highly valuable animal for preserving endangered species.

## Materials and methods

**Animals**

In this experimental prospective study 24 male 6-10 weeks Swiss white mice was purchased from Razi Institute (Tehran-Iran) and kept for 2 weeks in animal house in order to accommodate with its atmosphere. They were kept in 12 h light and 12 h dark. This study has been approved by ethic committee of Shahrekord University of Medical Sciences.

**Preparation of spermatozoa**

Male mice were killed by cervical dislocation. The animals were placed in refrigerator (4-6^o^C) immediately after their death for up to 12 days before the cauda epididymes were removed. On the 0 (control group), 1^st^, 2^nd^, 3^rd^, 5^th^, 7^th^, 10^th^ and the 12^th^ days cauda epididymides of 3 mice were removed and squeezed with a pair of forceps to release a dense mass of spermatozoa into a Petri dish containing 200 μl of Ham's F10 medium (Gibco BRL, Life Technologies, Ltd, Paisley, UK). Spermatozoa were suspended in Ham's F10 medium (7, 8).

**Sperm viability and total motility assessment**

The percentage of motile sperm cells was assessed with 40 magnification of objective lens under light microscope. At least 200 spermatozoa were counted and the percentage of motile sperm cells (progressive or not progressive) were counted. Viability of spermatozoa was examined by supravital staining method (9). A drop of sperm suspension was placed on a spot plate and mixed with one drop of 1% aqueous eosin Y solution (Sigma, USA). 

After 15 seconds, two drops of 10% aqueous nigrosin (Sigma, USA) solution was added and thoroughly mixed. A drop of this mixture was transferred to a clean glass slide. A thin smear was made and then air dried. The smears were examined with a 100 oil magnification. Live sperm cells appear white and dead sperms pink. At least 300 spermatozoa were counted and the result expressed as the percentage of live sperm.

**Sperm DNA integrity assessment**

For assessment of sperm DNA integrity, AO staining (10, 11) was used. In brief, for AO staining, a dried smear fixed in Carnoy's solution (Sigma Chemicals, St Louis, MO, USA) for at least 2 hours and air dried again. Then the sperm smears were stained with AO solution (0.02% AO in citrate-phosphate buffer, pH 2.5). After 5 minutes, the smear was washed with distilled water, covered with a coverslip and sealed with a nail polish to protect the smear from drying. Smears were examined using a fluorescence microscope (Olympus-Japan) with the following filter combination: 450-490 nm excitation, 510 nm reflector and 520 nm barrier filter.

The nuclei of 200-300 spermatozoa from each smear were examined and scored as green or red. Normal sperm heads show green tingle whereas denatured or single stranded DNA stains red and the result expressed as percentage of unchanged (green) nucleus sperms. AO stain intercalates into double stranded DNA as a monomer and binds to single stranded DNA as an aggregate. 

The monomeric AO bound to native (double stranded and normal) DNA fluoresces green, whereas the aggregated AO on denatured (single stranded) DNA fluoresces red (12). 

**Statistical analysis**

The data was analyzed using Chi-square test by Statistical Package for Social Studies (SPSS software version 12). A comparison was considered significantly different when p<0.05.

## Results

The viability, motility and DNA denaturation of mouse spermatozoa retrieved from cauda epididymes of dead animals at various days after their death are shown in table I. These sperm parameters were followed till 12 days after death (on the 0,1^st^, 2^nd^, 3^rd^, 5^th^, 7^th^, 10^th^ and the 12^th^ days).

**Sperm viability**

Viability had a decreasing trend from 0 till 12 days after death (p<0.001). At the 0 day about 87% of spermatozoa were alive whereas 12 days after death only 29% of sperms were still alive. There was no significant differences between viability percentages not only on the 0, 1^st^ and 2^nd^ days (p=0.42) but also between the 3^rd^ day and the days after it (p=0.80). 

However as it is shown in table I, there was significant differences between percentage of viable spermatozoa on the 0-2^nd^ days and the 3^rd^ to 12^th^ days (p<0.001). The viability of sperm cells decreased abruptly in the 3^rd^ day after death. 

**Sperm total motility**

As well as viability, total motility also had a decreasing trend from the 0 till the 12^th^ days after death (p<0.001). About 73% of spermatozoa were motile exactly after death (0 day) whereas about 14% of sperm cells were still motile after 12 days. There was not any significant differences between percentages of motility on the 0, 1^st^ and the 2^nd^ days (p=0.736), also between the 3^rd^ and the 5^th^ days (p=0.123). Moreover no significant differences was observed between the 7^th^, 10^th^ and 12^th^ days (p=0.288) (Table I). But as it is shown in Table 1 there was significant differences between percentages of motile spermatozoa on the 0 to the 2^nd^ days and the 3^rd^-5^th^ day after death (p<0.001). Also significant differences was seen between sperm motility percentages on the 3^rd^-5^th^ and 7^th^-12^th^ days after death (p<0.001). Motility of spermatozoa decreases abruptly on the 3^rd^ day after death .Another sudden decrease was seen at the 7^th^ day after death. 

**Sperms DNA integrity**

In contrast with viability and motility, percentage of sperm cells with native or double stranded DNA didn’t show any significant changes on the 0 to 12 days after death (p=0.99). Immediately after death (0 day) about 98% of spermatozoa were green (double stranded) and 12 days after death the same percentage (about 98% of spermatozoa) were still remained green (double stranded) (Table I).

**Table I:** Total motility, viability and DNA integrity of spermatozoa collected from mouse cadavers stored at 4-6˚C

**Days after death**	**Viability (%)** **(mean ± SD)**	**Total motility (%)** **(mean ± SD)**	**Intact nucleus (%)** **(mean ± SD)**
0	87 ± 2.2ª	73.3 ± 0.5^ c^	98 ± 0.8
1	82.6 ± 2.1 ª	71.7 ± 1.7^ c^	98.3 ± 0.5
2	79 ± 0.8 ª	70.7 ± 1.7^ c^	97.7 ± 0.5
3	46.6 ± 2.6^ b^	37.7 ± 2.1^ d^	98.7 ± 0.5
5	37.3 ± 6.8^ b^	30.3 ± 5.6^ d^	98.3 ± 0.5
7	29.4 ± 7.6^ b^	19.7 ± 1.7^ e^	98.7 ± 0.5
10	27.7 ± 8.6^ b^	18 ± 0.8^ e^	98 ± 0.6
12	29 ± 5.4^ b^	14.3 ± 2.5^ e^	98.7 ± 0.5

Each factor studied in different times after death up to 12 days. At each interval after death every factor was examined in three samples.

Different letters (a, b, c, d, e) within the columns indicates significant differences (p<0.05).

## Discussion

After the death of male animals, the spermatozoa within the testis and epididymides eventually disintegrate. Postmortem sperm recovery from the epididymides may constitute a powerful tool for the conservation of valuable genetic material and short term preservation of sperm cells without freezing can avoid decreasing of motility and fertility of some species (13, 14). Nichi *et al* showed that short storage of bull epididymides at 4^o^C provided sperm of higher quality and in vitro fertilization capacity than storage at 34^o^C (13). Fan *et al* stored mouse epididymides at 4^o^C in mineral oil or in mouse body for up to 4 days after death and retrieved spermatozoa were used to fertilize fresh oocytes. 

There was not any significant difference in fertilization rates when epididymides were stored for up to 2 days, but fertilization rates in mineral oil were higher than those in the mouse body at 3 and 4 days (15). Ganan *et al* have explored the effect of epididymis storage time at 5^o^C on sperm motility and percentage of intact acrosome in domestic cat. When epididymides were stored for 72 hours, best results are achieved when sperm are recovered from epididymides within 24 hours of cool storage (16). Moreover several studies suggest that preserving epididymidis of different animals (red deer, ram and boar) at 4 to 5˚C for several days can retain some sperm parameters (17-19).

Kishikawa *et al* maintained mouse cadavers in refrigerator at 4^o^C up to 20 days. They assessed motility and viability of spermatozoa only on the 0, 5^th^, 10^th^, 15^th^, 20^th^ day. About 30% of the spermatozoa collected 10 days after death were viable and when the spermatozoa were injected into oocytes, the fertilization rate was over 80% (4).

In the present study dead mice have been kept in refrigerator till 12 days after their death. The results show that motility and viability of mouse spermatozoa decrease progressively after their death. Moreover in this study these factors were examined on the 0, 1^st^, 2^nd^, 3^rd^, 5^th^, 7^th^, 10^th^ and 12^th^ day. The results show that on the 3^rd^ day after death the motility and viability decrease abruptly. This is the first time that such results are obtained. This shows that if epididymal spermatozoa of mouse cannot be collected immediately (when animal kept at 4-6^o^C), the motility and viability of spermatozoa are high until 48 hours after death. These sperm cells can be used for in vitro fertilization. This could be a good way for obtaining sperm cells from genetically valuable animals or endangered species.

Our results in mouse are similar to the results of Kikuchi *et al* on boar sperm cells (18). They suggests that cryopreservation of spermatozoa from boar epididymes stored at 4^o^C for 1-2 days, can be used for conserving male germ cells when epididymal spermatozoa cannot be collected immediately and cryopreserved.

Also Kaabi *et al* kept epididymidis of ram in 5^o^C and suggests when epididymal spermatozoa cannot be collected immediately; a good protocol for post-mortem semen collection is to process the samples in the first 24 h after the animal's death (19). On the basis of our results, it's apparent that motility and viability have a decreasing trend from 0-12 days after death when animal kept in refrigerator but surprisingly the proportion of spermatozoa with double stranded DNA remains unchanged. On the 12^th^ day after death 29% of spermatozoa are still alive, whereas the proportion of sperms with double stranded DNA is still 98%.

As mentioned before kishikawa *et al* have maintained mouse cadavers in refrigerator at 4^o^C (4). About 30% of the spermatozoa collected 10 days after death by them were viable, but they had limited ability to fertilize oocytes in vitro but when they injected the sperms into oocytes the fertilization rate was over 80%. In our study after 10 days only about 28% of sperms are still alive but about 98% of sperms still have double strande DNA.

Our findings can clearly explain the reason of fertilization of over 80% of oocytes when injected by spermatozoa that kept in the body of cadavers over 10 days in refrigerator. Although most of sperm cells 10 days after death of animal classified as dead, as determined by the live-dead stain, but they still have intact DNA and they are able to produce normal fertile offspring by ICSI as the Kishikawa *et al* report (4). Eventhough the sperm plasma membrane is disrupted, the sperm nuclei retain genotic integrity and are able to participate in embryo development.

This article is the first one that studies the proportion of double stranded DNA spermatozoa and shows that double stranded DNA sperm remains unchanged even 12 days after death when cadavers kept in refrigerator. If we have a look at the structure of sperm chromatin, there are great differences between sperm and somatic cell chromatin. Mammalian sperm DNA is the most tightly compacted eukaryotic DNA being at least six fold more highly condensed than the DNA in mitotic chromosomes. To achieve this high degree of packaging, sperm DNA interacts with protamines to form linear side-by-side arrays of chromatin. This compacted DNA can resist against different environmental and chemical factors (20). The results obtained from this study show that this structure can resist against denaturation too.

On this basis of this result sperm nucleus can resist against denaturation and it shows that also if environmental conditions are appropriate, some sperms with double stranded DNA is possible to find even in ancient animals like fossils. However this claim needs more investigation. Moreover resistance of sperm DNA against denaturation may help us to improve routine methods of sperm maintenance and to find new methods for the storage of spermatozoa like cryopreservation for a long period of time. These findings may be helpful for the progress of routine methods of preserving of spermatozoa. 

## Conclusion

Keeping of mouse spermatozoa in epididymis at 4-6^o^C lowers motility and viability of sperm cells but cannot lower the proportion of sperm cells with double stranded DNA. The sperm nucleus has the genetical information that is necessary for reproduction so by providing appropriate environmental condition sperm nucleus can resist against denaturation and maybe such nucleus can be used for assisted reproduction even in human cases. 

## References

[1] Christian N, Songsasen S, Leibo SP (1993). Presence of motile sperm in mice 24 hours postmortem. Theriogenology.

[2] Songsasen N, Tong J, Leibo SP (1998). Birth of live mice derived by in vitro fertilization with spermatozoa retrieved up to twenty-four hours after death. J Exp Zoology.

[3] An TZ, Wadas S, Edashige K, Sakurai T, Kasaei M (1999). Viable spermatozoa can be recovered from refrigerated mice up to 7 days after death. Cryobiology.

[4] Kishikawa H, Tateno H, Yanagimachi R (1999). Fertility of mouse spermatozoa retrieved from cadavers and maintained at 4 degree C. J Reprod Fertil.

[5] Kaneko T, Nakagata N (2005). Relation between storage temperature and fertilizing ability of freeze-dried mouse spermatozoa. Comp Med.

[6] Ward MA, Kaneko T, Kusakabe H, Biggers JD, Whittingham DG, Yanagimachi R (2003). Long-term preservation of mouse spermatozoa after freeze-drying and freezing without cryoprotection. Biol Reprod.

[7] Ben K, Hamilton MS, Alexander NJ (1988). Vasectomy-induced autoimmunity. Immunol.

[8] Mdhluli MC, van der Horst (2002). The effect of oleanolic acid on sperm motion characteristics and fertility of male Wistar rats. Lab Anim.

[9] Eliasson R (1977). Supravital staining of human spermatozoa. Fertil Steril.

[10] Tejada RI, Mitchell JC, Norman A, Marik JJ, Friedman S (1984). A test for the practical evaluation of male fertility by acridine orange (AO) fluroscence. Fertil Steril.

[11] Varghese AC, Bragias FM, Mukhopadhyay D, Kundu S, Pal M, Bhattacharyya AK (2009). Human sperm DNA integrity in normal and abnormal semen samples and its correlation with sever Sperm characteristics. Andrologia.

[12] Hoshi K, Katayose H, Yanagid K, Kimura Y, Sato A (1996). The relationship between acridine orange fluorescence of sperm nuclei and the fertilizing ability of human sperm. Fertil Steril.

[13] Nichi M, Goovaerts IGF, Cortada CNM, Barnabe VH, De Clercq JBP, Bols PEJ (2007). Roles of lipid proxidation and cytoplasm droplets on invitro fertilization capacity of sperm collected from bovine epididymides stored at 4 and 34 degree C. Theriogenology.

[14] Kaneko T, Fukumoto K, Haruguchi Y, Kondo T, Machida H, Koga H (2009). Fertilization of C5713L/6 mouse sperm collected from cauda epididymides after preservation or transportation at 4 degree C using laser-microdissected oocytes. Cryobiology.

[15] Fan ZQ, Li XW, Liu Y, Meng QG, Wang YP, Hou YP (2008). Piezo-assisted in vitro fertilization of mouse oocytes with spermatozoa retrieved from epididymides stored at 4 degree C. J Reprod Dev.

[16] Ganan N, Gomendio M, Roldan ERS (2009). Effect of storage of domestic cat (Felis Catus) epididymides at 5 degree C on sperm quality and cryopreservation. Theriogenology.

[17] Soler AJ, Perez-Guzman MD, Gard JJ (2003). Storage of red deer epididymides for four days at 5 degrees C: effects on sperm motility, viability and morphological integrity. J Exp Zoology A Com Exp Biol.

[18] Kikuchi K, Nagai T, Kashiwazaki N, Ikeda H, Noguchi J, Shimada A (1998). Cryopreservation and ensuing in vitro fertilization ability of boar spermatozoa from epididymides stored at 4 degree C. Theriogenology.

[19] Kaabi M, Paz P, Alvarez M, Arel E, Boixo JC, Rouissi H (2003). Effect of handling conditions on quality of ram spermatozoa recovered post-mortem. Theriogenology.

[20] Ward WS, Coffey DS (1991). DNA packaging and organization in mammalian spermatozoa: comparison with somatic cells. Biol Reprod.

